# Pancreatic pseudocyst with biliary fistula diagnosed using a novel slim peroral cholangioscope

**DOI:** 10.1055/a-2253-8912

**Published:** 2024-02-15

**Authors:** Haruo Miwa, Kazuya Sugimori, Kazuki Endo, Ritsuko Oishi, Hiromi Tsuchiya, Takashi Kaneko, Shin Maeda

**Affiliations:** 1 Gastroenterological Center, Yokohama City University Medical Center, Yokohama, Japan; 2 Department of Gastroenterology, Yokohama City University Graduate School of Medicine, Yokohama, Japan

A novel slim cholangioscope (eyeMax 9-Fr; Micro-Tech, Nanjing, China) has been developed in recent years 1 2 . We report a case of pancreatic pseudocyst with biliary fistula diagnosed using this novel cholangioscope ( Video 1 ).

A novel slim cholangioscope, 9-Fr eyeMax (Micro-Tech. Nanjing, China), was useful for the diagnosis of pancreatic pseudocyst with biliary fistula.Video 1

An 88-year-old man was referred to our hospital because of multiple bile duct stones with biliary stricture. Magnetic resonance imaging and computed tomography showed a large cystic lesion at the pancreatic head ( Fig. 1 , Fig. 2 ). We performed endoscopic retrograde cholangiopancreatography for stone removal and diagnosis of the stricture.

**Fig. 1:**
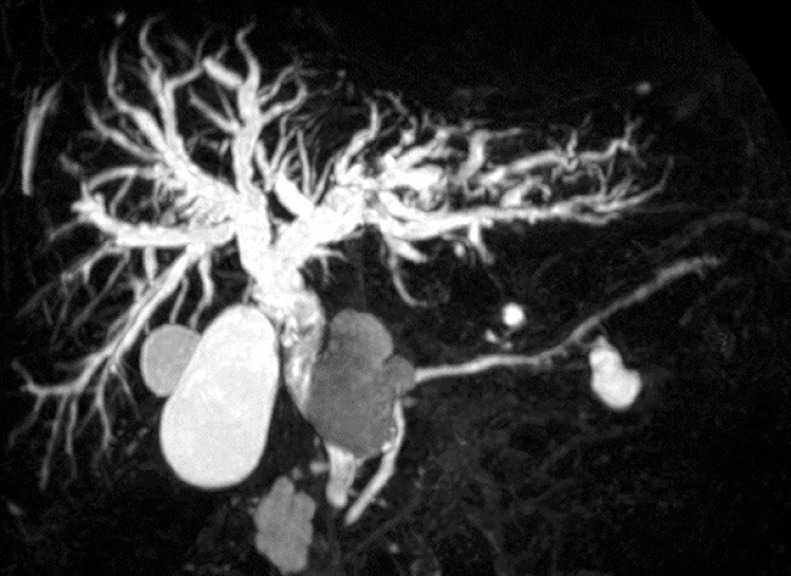
Magnetic resonance imaging showed a large cystic lesion at the pancreatic head and multiple stones at the perihilar bile duct.

**Fig. 2:**
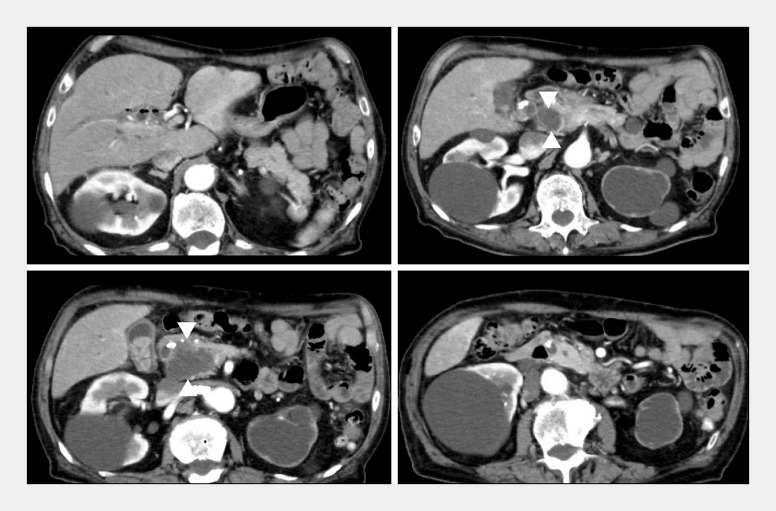
Computed tomography showed that bile duct dilation was improved by a biliary stent. However, the pancreatic cyst had not decreased in size (arrowheads).

Initial cholangiography showed the compressed distal bile duct and multiple stones filling the perihilar bile duct. A guidewire was advanced into the cyst lesion, and a fistula between the bile duct and pancreatic cyst was confirmed ( Fig. 3 ). EyeMax (9-Fr) was inserted to evaluate the stricture and fistula ( Fig. 4 ). The cholangioscope was easily advanced through the stricture, and multiple bile duct stones were observed. As the stricture was not too narrow, stone removal was attempted. Stones were removed using a basket catheter after endoscopic papillary large balloon dilation. The cholangioscope was reinserted, and complete stone removal was confirmed. Subsequently, a guidewire was placed into the pancreatic cyst through the biliary fistula, and the cholangioscope was advanced toward the guidewire. Cystography revealed no connection between the cyst and pancreatic duct. Findings suggestive of a neoplastic lesion were not observed in the inner wall of the cyst. Biopsy of the cyst wall and the biliary fistula was performed via direct viewing. Pathological results showed no epithelial tissues; therefore, the diagnosis was a pancreatic pseudocyst with a biliary fistula. The cyst decreased in size during the month following stone removal.

**Fig. 3:**
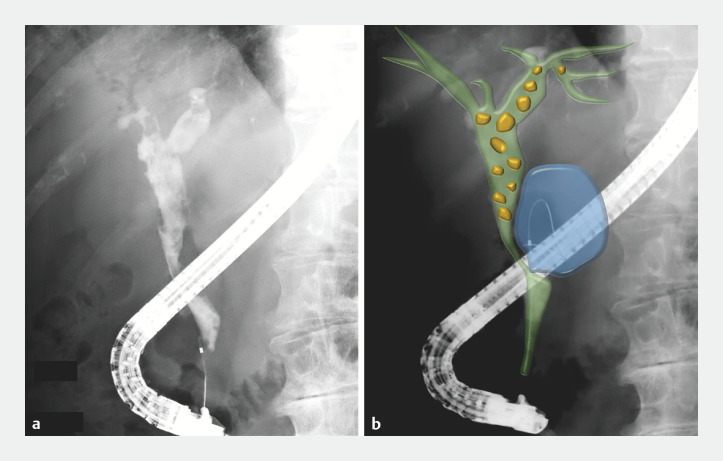
Cholangiography images. **a** Cholangiography showed a compressed distal bile duct. **b** A guidewire was advanced into the cyst, revealing the fistula.

**Fig. 4:**
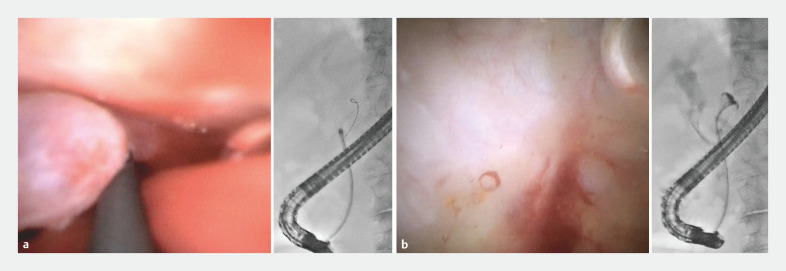
Cholangioscopy images. **a** The cholangioscope was advanced into the cyst through the biliary fistula. **b** Findings suggestive of a neoplastic lesion were not observed in the inner wall of the cyst.

Pancreatic pseudocyst with biliary fistula is an extremely rare condition 3 4 5 . To the best of our knowledge, this is the first report of the fistula being confirmed using cholangioscopy.

Endoscopy_UCTN_Code_CCL_1AZ_2AH

## Notes

Endoscopy E-Videos https://eref.thieme.de/e-videos
*E-Videos* is an open access online section of the journal *Endoscopy* , reporting on interesting cases and new techniques in gastroenterological endoscopy. All papers include a high-quality video and are published with a Creative Commons CC-BY license. Endoscopy E-Videos qualify for HINARI discounts and waivers and eligibility is automatically checked during the submission process. We grant 100% waivers to articles whose corresponding authors are based in Group A countries and 50% waivers to those who are based in Group B countries as classified by Research4Life (see: https://www.research4life.org/access/eligibility/ ). This section has its own submission website at https://mc.manuscriptcentral.com/e-videos .
